# Russian Regional Differences in Allele Frequencies of CFTR Gene Variants: Genetic Monitoring of Infertile Couples

**DOI:** 10.3390/genes15010045

**Published:** 2023-12-27

**Authors:** Andrey S. Glotov, Vyacheslav B. Chernykh, Olga A. Solovova, Aleksander V. Polyakov, Maksim Yu. Donnikov, Ludmila V. Kovalenko, Yury A. Barbitoff, Yulia A. Nasykhova, Tatyana E. Lazareva, Oleg S. Glotov

**Affiliations:** 1 D.O. Ott Research Institute of Obstetrics, Gynaecology, and Reproductology, 199034 St. Petersburg, Russiayulnasa@gmail.com (Y.A.N.); olglotov@mail.ru (O.S.G.); 2 Research Centre for Medical Genetics, Moskvorechie Street 1, 115522 Moscow, Russia; 3 Budgetary Institution of Highest Education of KHMAO-Yugra “Surgut State University”, 628400 Surgut, Russia; donnikov@gmail.com (M.Y.D.);

**Keywords:** allele frequency, CFTR gene, genetic monitoring, male infertility, genetic variants

## Abstract

A male factor, commonly associated with poor semen quality, is revealed in about 50% of infertile couples. *CFTR* gene (Cystic Fibrosis Transmembrane Conduction Regulator) variants are one of the common genetic causes of azoospermia-related male infertility. Notably, the spectrum and frequency of pathogenic *CFTR* variants vary between populations and geographical regions. In this work, we made an attempt to evaluate the allele frequency (AF) of 12 common CFTR variants in infertile Russian men and healthy individuals from different districts of Russia. Because of the limited number of population-based studies on Russian individuals, we characterized the population AFs based on data from the Registry of Russian cystic fibrosis (CF) patients. In addition to the CF patient registry, we estimated the local frequencies of the same set of variants based on the results of genotyping of CF patients in local biocollections (from St. Petersburg and Yugra regions). AFs of common CFTR variants calculated based on registry and biocollection data showed good concordance with directly measured population AFs. The estimated region-specific frequencies of CFTR variants allowed us to uncover statistically significant regional differences in the frequencies of the F508del (c.1521_1523del; p.Phe508del) and CFTRdele2,3(21kb) (c.54-5940_273+10250del21kb; p.Ser18ArgfsX) variants. The data from population-based studies confirmed previous observations that F508del, CFTRdele2,3(21kb), and L138ins (c.413_415dup; p.Leu138dup)variants are the most abundant among infertile patients, and their frequencies are significantly lower in healthy individuals and should be taken into account during genetic monitoring of the reproductive health of Russian individuals.

## 1. Introduction

Infertility is a major issue for human reproductive health, affecting about 15% of couples worldwide, and the contribution of male and female factors are approximately equal [1]. Various genetic factors are known to be involved in the etiology of male infertility, including: (i) sex chromosome aneuploidies and mosaicism, (ii) balanced chromosome rearrangements, (iii) Y chromosome microdeletions in the azoospermia factor (*AZF*) locus and other copy number variants (CNVs), (iv) pathogenic variants in many genes (*AR*, *ATXN1*, *CPOLG*, *DMPK*, *SHBG*, *TEX11*, and others), (v) dysregulated miRNAs, and (vi) altered DNA and histones methylation, as well as other genetic and epigenetic factors [2,3].

One of the most studied genetic causes of male infertility is Cystic Fibrosis Transmembrane Conduction Regulator (*CFTR*) gene variants [4]. Pathogenic *CFTR* gene variants are a cause of cystic fibrosis (CF, OMIM#219700), and CFTR-related disorders (CFTR-RD). Pathogenic variants of the *CFTR* gene can result in a congenital bilateral absence of the vas deferens, which could be an isolated syndrome (Congenital Bilateral Aplasia (absence) of the Vas Deferens (CBAVD, OMIM# 277180)) or a clinical feature of CF, leading to obstructive azoospermia in 95% of males with CF [5,6]. Moreover, associations between *CFTR* variants and other forms of male infertility, namely non-obstructive azoospermia and oligozoospermia, were revealed in a recent meta-analysis [7].

According to the locus-specific CFTR2 database, about 800 variants discovered in the *CFTR* gene have been classified based on their clinical significance (https://cftr2.org, accessed on 29 November 2023). The frequency and spectrum of the *CFTR* variants vary significantly between different countries and ethnic groups. F508del (c.1521_1523del; p.Phe508del) is known as the most frequent pathogenic *CFTR* variant both worldwide and in the Russian Federation [8,9,10,11]. Other frequent CF-causing variants in the Russian population are CFTRdele2,3(21kb) (c.54-5940_273+10250del21kb; p.Ser18ArgfsX16), E92K (c.274G>A; p.Glu92Lys), 1677delTA (c.1545_1546delTA; p.Tyr515Ter), 3849+10kbC>T (c.3718-2477C>T), 2143delT (c.2012del; p.Leu671Ter), W1282X (c.3846G>A; p.Trp1282Ter), and N1303K (c.3909C>G; p.Asn1303Lys) [9,11]. Remarkably, despite the high prevalence of obstructive azoospermia in individuals with CF, prior investigations have demonstrated distinct spectrum and frequencies of *CFTR* variants among CF patient cohorts compared with those with CBAVD without CF. In a cohort of infertile Russian men without CF, the most commonly identified variants were F508del, CFTRdele2,3(21kb), and L138ins (c.413_415dup; p.Leu138dup) [12].

Distinct *CFTR* variant profiles in CF and CBAVD individuals, as well as in other genes in which variants may not only cause monogenic disorders but could also be risk factors for complex traits, underscore the importance of genetic monitoring, enabling us to tailor population-specific preventive measures and genetic counseling strategies accordingly. The main and most promising data source for population allele frequencies of genetic variants linked to hereditary diseases, especially monogenic ones, are biobank-scale genetic datasets [13]. Allele frequency (AF) data for variants causing monogenic diseases are important for two main reasons: (i) for making more precise decisions regarding disease diagnostics in affected families and (ii) for the development of region- or ethnicity-specific measures for the diagnosis and prevention of inherited diseases. The identification of variants that also could be risk factors for complex traits such as reproductive failures may be important for evaluating genetic risks for couples, as well as for predicting specific reproductive outcomes. The implementation of reproductive genetic risk assessment in the general population, especially for those planning in vitro fertilization (IVF) and intracytoplasmic sperm injection (ICSI) procedures, will aid in early (prior to conception) carrier identification.

At present, pathogenic variants in the *CFTR* gene, along with other genes causing most common autosomal recessive (CF, phenylketonuria, spinal muscular amyotrophy, and others) and common X-linked recessive diseases (Duchenne/Becker muscular dystrophy, hemophilia A and B, and others), are actively screened in fertile individuals and couples, as well as in patients planning to undergo IVF/ICSI. However, the efficiency of current *CFTR* testing practices is sub-optimal, often due to overlooking ethnic issues and AF differences among populations of distinct regions.

Our work seeks to assess the frequency of pathogenic *CFTR* variants identified in infertile men in Russian regions based on data from population studies and CF studies, including the results of individuals with CF genotyping and data from the Registry of Russian CF Patients.

## 2. Materials and Methods

We used genetic data from several independent sources. The allele frequency (AF) of common pathogenic *CFTR* variants was estimated using two approaches: (i) direct AF measurements in the healthy population and (ii) indirect calculation of AF based on the spectrum of variants in CF patients.

### 2.1. Direct Measurements of CFTR Variant Frequencies

The RUSeq database was taken as the first source [14]. Today, this database is the only publicly available source of AFs in the Russian population. RUSeq integrates genetic information from major medical genetic laboratories in the Russian Federation, and the current dataset includes as many as 7492 exome samples collected from two of the biggest Russian cities (Moscow and St. Petersburg). For our study, we utilized the database to obtain information about AFs in healthy individuals (n = 1671). However, RUSeq lacks comprehensive data on AFs across Russia’s Federal districts. Therefore, additional data sources were employed for comparison.

In addition to RUSeq data, the frequencies of common *CFTR* variants estimated earlier by the Research Centre for Medical Genetics were included in the study to characterize the Moscow region (n = 1327) [9,11]. To characterize the frequency and spectrum of *CFTR* variants among infertile Russian men, the cohort from a previous study was incorporated into the current research (n = 6033) [6].

### 2.2. Indirect Calculation of CFTR Variants Frequencies from Epidemiological Information

The indirect calculation of AF for common *CFTR* variants was based on two input parameters: (i) information on the proportion of a given variant among CF patients and (ii) the overall prevalence of CF in the population. Data on the CF prevalence were taken from the Registry of Russian CF patients (n = 3298) [15]. For the first step of the procedure, we used the registry data to calculate the prevalence of CF in each federal region of the Russian Federation (Supplementary Figure S1). This was calculated as follows

(1) $$F_{d}=\frac{n}{N\times 18},$$

where *n* is the number of children with CF under the age of 18 in the region and *N* is the average number of newborns in the region in the last 18 years prior to the year of the Registry’s publication (2021). The expected proportion of pathogenic variant carriers (*F_c_*) was then calculated from the disease prevalence assuming the Hardy–Weinberg equilibrium, as follows:

(2) $$F_{c}=\sqrt{F_{d}},$$

We then calculated the expected regional AF of each variant by multiplying the frequency of the disease allele carriers (*F_c_*) by the frequency of the allele among CF patients in this region (f_a_). The f_a_ values were either taken from the same CF patient registry (for each of the federal districts) or calculated using the data from the database of the multicenter research bioresource collection of the “Human Reproductive Health” project (for St. Petersburg (n = 2412) and the Yugra region (n = 54)). This database aggregates the results of the genotyping for patients with hereditary and reproductive diseases.

### 2.3. Statistical Analysis and Code Availability

A chi-squared test was employed to investigate the disparities in allele frequency between the studied cohorts. All data and code pertinent to the analysis presented in this work are available at https://github.com/tanya-lazareva/cftr_disctricts.git (accessed on 24 December 2023).

## 3. Results

To conduct a comparative analysis, we chose 12 *CFTR* gene variants that are common among infertile Russian men, based on a previous study of Chernykh et al.: F508del, CFTRdele2,3(21kb), L138ins, W1282X, 1677delTA, 3849+10kbC>T, E92K, 2143delT, G542X (c.1624G>T; p.Gly542Ter), 2184insA (c.2052dup; p.Gln685ThrfsTer4), N1303K, and R334W (c.1000C>T; p.Arg334Trp) [6]. We began by examining the AF of the selected variants in population-based studies (the RUSeq database and the study by Petrova et al. [11]). Eleven out of twelve selected variants were directly detected in these sources. The only missing variant was the 2184insA insertion, which was not identified in either dataset. The F508del allele appeared as the most common one, corroborating previous findings [8,10], while the second most frequent in the European part of Russia was the CFTRdel2,3(21kb) allele. Despite the high carrier rates, the frequencies of these *CFTR* variants in infertile men were found to be approximately two times higher compared with the healthy RUSeq cohort and population study of Petrova et al. (F508del: chi-squared = 9.5131, df = 1, *p*-value = 0.00204; CFTRdel2,3(21kb): chi-squared = 37.231, df = 1, *p*-value = $1.05\times 10^{-9}$) [11]. The L138ins variant frequency showed the same trend (see Table 1). Interestingly, the total frequency of the 11 variants was also substantially higher among infertile men compared with either source of population AFs (0.0240 compared to the maximum value of 0.0117 for the healthy individuals from RUSeq), but lower in infertile Russian men without CBAVD syndrome (AF = 0.0164) (chi-squared = 100.19, df = 2, *p*-value < $2.2\times 10^{-16}$). This finding corroborated previous studies [12], reinforcing the significance of *CFTR* gene variants in male infertility monitoring. However, due to the low carrier rate of other variants in the population data, definitive conclusions regarding these variants could not be drawn.

While population-based studies are an important source of AF information, they are underpowered when estimating the differences in AFs of *CFTR* variants in underrepresented regions of Russia. An alternative approach could be utilizing well-characterized cohorts of individuals with CF for monitoring the spectrum and frequency of *CFTR* variants in the context of infertility assessments. It is important, however, to validate that the AF estimates based on the CF patient cohort and epidemiological information were accurate enough to enable further analysis. To conduct such a validation, we compared the frequency of the most common F508del variant calculated based on data derived from individuals with CF (see Section 2) with that obtained from population-based data (RUSeq database) (AF = 0.0054 vs. AF = 0.0080, 95% confidence interval 0.0053–0.0117). The negligible difference between these frequencies supported the use of the CF patient cohort for evaluating regional variation in *CFTR* variant frequencies across Russian Federal districts. Given this finding, we then estimated the frequencies of all *CFTR* variants studied in the eight federal districts or Russia (Supplementary Table S1, Supplementary Figure S1). In most regions, the F508del and CFTRdele2,3(21kb) variants were the most common, with the North Caucasus and Volga Federal District being the only exception, where the most abundant alleles among individuals with CF were 1677delTA and F508del (for the North Caucasus) and F508del and E92K (for the Volga district). To validate the significance of the observed differences, we statistically compared the proportions of different CF-causing alleles across districts. Because of the limited sample size and the inability to carefully evaluate the variation in the spectrum of rare *CFTR* variants across distinct federal regions, we combined all of the observed variant frequencies into a single “other” group, except for the two most abundant variants based on the registry data, namely F508del and CFTRdele2,3(21kb). The statistical analysis confirmed the presence of the significant differences in the *CFTR* variant spectrum (chi-squared = 197.55, df = 14, *p*-value < $2.2\times 10^{-16}$). The highest frequency of the CFTRdele2,3(21kb) variant was revealed in the Central Federal Region. The overall abundance of “other” variants was significantly higher in the North Caucasus Federal District (AF = 0.7706), which aligns with previous studies [11]. In contrast, the prevalence of F508del and CFTRdele2,3(21kb) among the causal variants of CF was shown for the population from Siberia (Table 2).

Analysis of AFs based on the results of genotyping in individuals with CF from St. Petersburg (part of the Northwestern Federal District) and the Yugra region (part of the Ural Federal District) provided additional insights into the fine-scale differences in variant frequencies within federal districts. It was shown that F508del was a more prevalent allele in St. Petersburg (chi-squared = 15.238, df = 1, *p*-value = $9.5\times 10^{-5}$), and variants other than F508del (chi-squared = 14.065, df = 2, *p*-value = 0.0008827) were more abundant in Yugra (Table 2). Interestingly, this difference did not appear when comparing the Northwestern and Ural Federal Districts, respectively, likely due to the aggregation of data from ethnically diverse regions that formed parts of the larger districts. This reinforces the value of integrating local biosample collections and data collection to gain a more comprehensive understanding of genetic variation within a population.

## 4. Discussion

Genetic monitoring is crucial for assessing the genetic load in a population through evaluating distribution of the frequencies of variants in genes linked to monogenic diseases. Besides estimating the risk of monogenic disease, genetic monitoring could provide important information regarding the genetic causes of infertility. For example, pathogenic *CFTR* variants result in CF and CFTR-RD, but are also associated with male infertility. Thus, by analyzing the AFs of variants of the *CFTR* gene in the general population, one could gain valuable insight into genetic factors affecting male fertility. Additionally, this approach enables us to accurately identify carriers and propose IVF/ICSI programs with preimplantation genetic testing of monogenic disorders (PGT-M) for such couples, helping to reduce the risk of CF in offspring.

Currently, Russia lacks a national large-scale database of genetic variation. However, multiple local databases of human exome sequencing, registers of some hereditary diseases, and network sources of genetic information are being created based on data from biobank genetic collections. RUSeq is the largest resource of genetic variation data for the Russian population [14]; however, even this resource has known issues as a result of uneven coverage of different regions of Russia, leading to fuzzy estimates of AFs for local subpopulations. To overcome the limitation of using RUSeq as a single source, in this study, we tried to use additional laboratory-based databases and a CF patient registry to estimate the AF of pathogenic *CFTR* variants that are common in both CF and infertile male patients; hence, to evaluate these sources as tools for estimating the local AFs. As a result of this analysis, several important tendencies were revealed.

Our initial investigations revealed that utilizing registry data to approximate population AFs yielded comparable results to directly measured AFs in the Russian population. However, this approach yielded slightly lower AF estimates compared with direct observations in the general population. This discrepancy might be attributed to an incomplete representation of the CF cohort in the registry data. Nonetheless, the registry’s focus on large districts rendered it incapable of identifying the fine-scale variation in AF within the regions. To precisely identify these differences, the establishment of local biocollections, as demonstrated in the present study for St. Petersburg and the Yugra region, could provide promising solutions.

It was also shown that most frequent variants, namely F508del and CFTRdele2,3(21kb), must obligatorily be included for genetic screening in infertile Russian men because of the significantly higher AF of these variants in these patients compared with the general Russian population (see Table 1). These *CFTR* variants were the most common in six out of eight of Russia’s Federal districts. The situation with other rare variants seemed less clear due to insufficient sample sizes for making solid conclusions regarding the frequencies of rare variants. For instance, the CF registry data proved insufficient to accurately assess the allele frequencies of L138ins, observed in infertile Russian men and R117H (c.350G>A, p.Arg117His), which was identified at a higher frequency in cohort of infertile men in Western Europe [16]. This limitation stems from the registry’s relatively limited case numbers. Additionally, 5T/7T/9T is known to be associated with non-obstructive azoospermia and oligozoospermia [17,18].

Indeed, our study is preliminary in nature and has a large number of limitations related to the available genetic databases, information included therein, methods of genotyping, and other factors. Nevertheless, this approach demonstrates certain results and has the prospect of further development.

## 5. Conclusions

Extensive population databases and national registries of monogenic disorders are indispensable tools for assessing genetic risks and accurately predicting the reproductive outcomes associated with the *CFTR* gene. Additionally, regional genetic biobanks encompassing cohorts from various regions serve as valuable resources for establishing population differences in the spectrum and frequency of genetic variants. In the present study statistically significant variation in the frequencies of F508del, CFTRdele2,3(21kb) alleles were revealed across different Russian Federal Districts. It was also shown that three *CFTR* variants (F508del, CFTRdele2,3(21kb), and L138ins) are prevalent among infertile Russian males, highlighting their significance in genetic population monitoring in Russia.

## Figures and Tables

**Table 1:** Allele frequency (AF) of 12 common pathogenic CFTR gene variants in Russian infertile men and general populations in various regions of Russian Federation.

CFTR Gene Variant	rsID	RUSeq Healthy Population (n = 1671)	AF of the CFTR Variants in European Part of Russia (n = 1327) ^1^	AF of the CFTR Variants in Infertile Russian Men (n = 6033) ^2^	AF of the CFTR Variants in Infertile Russian Non-CBAVD Men (n = 5098) ^2^	Russian Federation Calculated AF ^3^
F508del	rs113993960	0.008	0.0057	0.0152	0.0113	0.0054
CFTRdele2,3(21kb)	-	n.a.	0.0004	0.0017	0.0011	0.0006
L138ins	rs397508686	0.0006	0	0.0015	0.0009	0.0002
W1282X	rs77010898	0.0006	0.0011	0.0013	0.0009	0.0002
1677delTA	rs121908776	0	n.a.	0.0007	0.0005	0.0002
3849+10kbC>T	rs75039782	0.001	n.a.	0.0007	0.0006	0.0002
E92K	rs121908751	0	0	0.0007	0.0004	0.0004
2143delT	rs121908812	0.0003	n.a.	0.0006	0.0002	0.0002
G542X	rs113993959	0.0003	n.a.	0.0005	0.0003	0.0002
2184insA	rs121908746	n.a.	n.a.	0.0005	0.0002	0.0002
N1303K	rs80034486	0.0009	n.a.	0.0003	0.0001	0.0002
R334W	rs121909011	0	n.a.	0.0003	0.0003	0.0001
Total frequency		0.0117	0.0072	0.0240	0.0164	0.0081

^1^—cohort studies include data of the Russian CFTR registry of individuals with CF considering the frequency of hereditary disease in the region [15]; ^2^—Data from study of Cherhykh et al. [6]; ^3^—AF calculated based on Russian CFTR registry of individuals with CF considering the frequency of hereditary disease in the region [15].

**Table 2:** Allele frequency (AF) of 12 common pathogenic CFTR gene variants in cohorts of individuals with CF in Russia’s Federal districts.

	F508del		CFTRdele2,3(21kb)		Other	
	AF	AC	AF	AC	AF	AC
Russian Federation (n = 3292) ^1^	0.5155	3400	0.0611	403	0.423	2793
Central Federal District (n = 805) ^1^	0.521	839	0.078	126	0.401	645
Northwestern Federal District (n = 263) ^1^	0.561	295	0.049	26	0.39	205
Southern Federal District (n = 323) ^1^	0.553	357	0.064	47	0.384	248
North Caucasian Federal district (n = 170) ^1^	0.214	73	0.016	5	0.771	262
Volga Federal District (n = 566) ^1^	0.504	571	0.052	59	0.444	502
Ural Federal District (n = 245) ^1^	0.538	264	0.048	24	0.412	202
Siberian Federal District (n = 362) ^1^	0.554	401	0.064	46	0.383	277
Far Eastern Federal District (n = 146) ^1^	0.536	157	0.069	20	0.394	115
St.Petersburg (n = 2412) ^2,3^	0.59	2846	0.037	178	0.373	1799
Yugra region (n = 54) ^2^	0.398	43	0.046	5	0.556	60

^1^—Data about individuals with CF under 18 years old from Russian CFTR registry of individuals with CF considering the frequency of hereditary disease in the region [15]; ^2^—Own data includes the results of patients genetic testing of laboratory cohorts of the region using NGS or/and PCR technology; ^3^—Cohort includes individuals with CF under 18 years old as well as adults.

## Data Availability

The code pertinent to the analysis presented in the current work are available at https://github.com/tanya-lazareva/cftr_disctricts.git (accessed on 24 December 2023).

## Supplementary Materials

The following supporting information can be downloaded at: https://www.mdpi.com/article/10.3390/genes15010045/s1, Supplementary Table S1: Allele frequency of variants discovered in infertile Russian men among CF cohorts according to Registry of Russian CF Patients; Supplementary Figure S1: Location of studied cohorts from the Registry of Russian CF Patients and local biocollections.

## Abbreviations

The following abbreviations are used in this manuscript:

AF	Allele frequency
CF	Cystic fibrosis
CF-RD	CFTR-related diseases
CBAVD	Congenital Bilateral Aplasia (absence) of Vas Deferens
