# Supplementary material for: Russian Regional Differences in Allele Frequencies of CFTR Gene Variants: Genetic Monitoring of Infertile Couples
Source: Genes (Basel). 2023 Dec 27;15(1):45. doi: 10.3390/genes15010045 (PMC10815393; doi:10.3390/genes15010045)
Supplement: Supplementary file 1 [file genes-15-00045-s001.zip › genes-2718947-supplementary/Supplementary Figure S1.pdf]

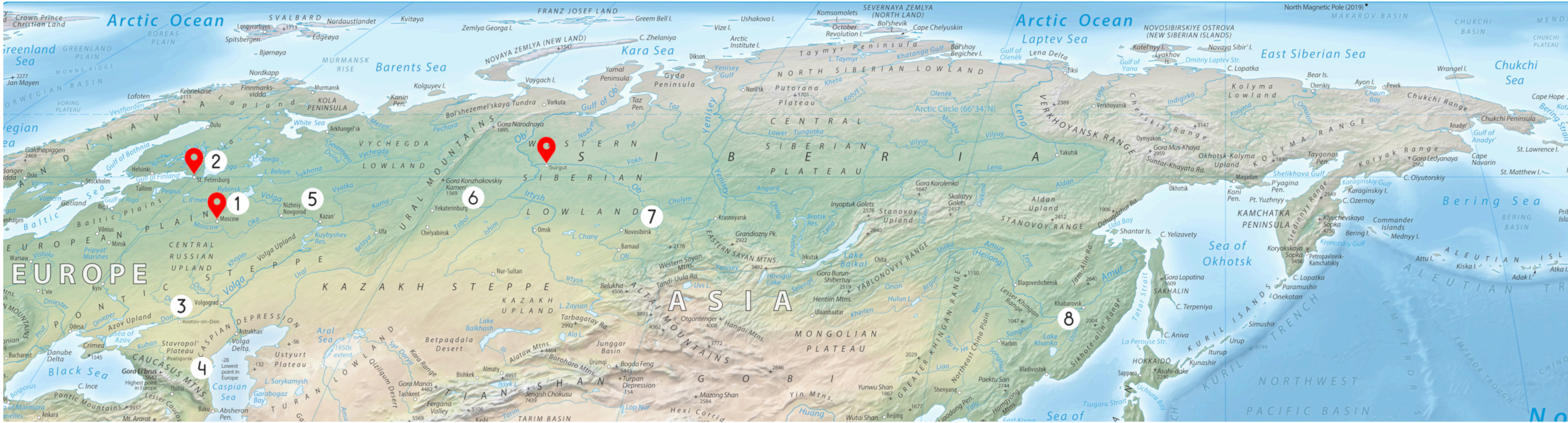

Administrative centre of
1 Central Federal District
2 Northwestern Federal District
3 Southern Federal District
4 North Caucasian Federal district
5 Volga Federal District
6 Ural Federal District
7 Siberian Federal District
8 Far Eastern Federal District

Location of local biocollection
